# Does the Selection of the Procedure Impact the Return to Work in Unemployed Patients Undergoing Bariatric Surgery?

**DOI:** 10.1007/s11695-022-06164-z

**Published:** 2022-07-04

**Authors:** Kevin Claassen, Kathrin Kügler, Jörg Celesnik, Metin Senkal, Pia Jäger

**Affiliations:** 1 Stiftungsprofessur Für Arbeitsmedizin Und Betriebliches Gesundheitsmanagement, Universität Witten/Herdecke, Stockumer Str. 10, 58453 Witten, Germany; 2 Klinik Für Orthopädie Und Spezielle Orthopädische Chirurgie, Lipper Weg 11, 45770 Marl, Germany; 3 Klinik Für Allgemein- Und Viszeralchirurgie, Charité Universitätsmedizin Berlin, Campus Benjamin Franklin, Hindenburgdamm 30, 12203 Berlin, Germany; 4 Klinik Für Allgemein- Und Viszeralchirurgie, Knappschaftskrankenhaus Bottrop, Osterfelder Straße 157, 46242 Bottrop, Germany; 5 Klinik Für Allgemein- Und Viszeralchirurgie, Marien Hospital Witten, Marienplatz 2, 58452 Witten, Germany

**Keywords:** Return to work, Unemployment, Sleeve gastrectomy, Roux-en-Y gastric bypass

## Abstract

**Purpose:**

Obesity and its comorbidities are risk factors for absenteeism and unemployment. Bariatric surgery might help to intervene in the vicious circle of unemployment, social disadvantage and increasing obesity. The most common bariatric procedures are sleeve gastrectomy (SG) and Roux-en-Y gastric bypass (RYGB). This survey analyzes the influence of the bariatric procedure on return to work.

**Methods:**

The data of a German nationwide multicenter registry StuDoQ|MBE from 2015 to 2020 are evaluated. Patients are surveyed who underwent a primary SG or RYGB while being unemployed: 782 patients are included. Primary endpoint is any form of return to work within 1 year after treatment. The surgical procedure acts as the binary main treatment variable. A multivariate binary logistic regression model was performed with age, sex, vocational training and weight loss as third variables so that odds ratios (OR) and adjusted ORs were determined.

**Results:**

Of the patients, 41.56% received a RYGB, 58.44% a SG. One year after bariatric surgery, 39.39% of the patients with SG and 33.85% with RYGB reached a return to work. The OR for return to work is 1.27 (*p* = 0.11) non-significant in favor of SG. The adjusted OR is 1.26 (*p* = 0.15), indicating that there is no significant influence of the difference between the two surgical procedures on the outcome of return to work.

**Conclusion:**

There is a positive effect regarding return to work in bariatric patients: More than a third of the previously unemployed patients were employed 1 year after surgery. Procedure-specific influences could not be determined.

**Graphical abstract:**

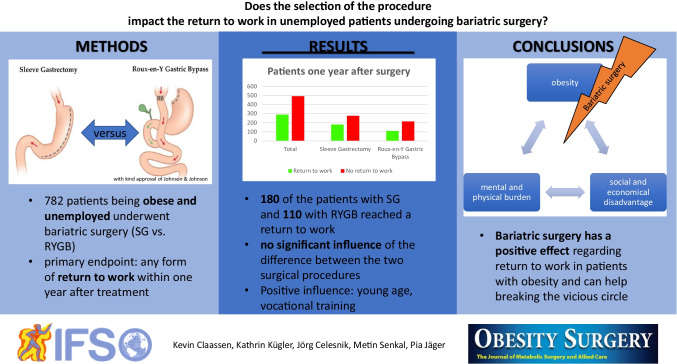

## Introduction

Obesity is a disease, which is able to induce several comorbidities, limit mobility and well-being as well as work productivity. It can also act as a cause of absenteeism or even unemployment. International studies found different conclusions on the question whether a bariatric surgery could improve the ability to work. While between 1975 and 2016, the prevalence of obesity almost tripled worldwide, the prevalence of obesity is 23.9% in the male and 23.3% in the female population in Germany. [1] [2] [3] In Germany, mental disorders and musculoskeletal disorders that occur more often in people with obesity are the most common health-related causes for absenteeism in work being together responsible for 38.3% of all incapacity days in 2019. [4] [5] [6] [7]

The Organisation for Economic Co-operation and Development (OECD) summed up the negative effect for people with obesity on the labor market as evidence from multiple studies (2007–2013) in their Health Working Paper of 2015:

- “Obese people are less likely to be employed than normal-weight persons.
- Obese people earn (up to 18%) less than non-obese, even when they have equivalent positions and discharge the same tasks.
- Obese people are less productive due to more days of sick leave, longer work absence and reduced performance while at work.” [8]

Both in the United States of America (USA) and in the European Union (EU), significant effects regarding the hourly wages were found in the adjusted analysis: In the USA, this effect was found to be distinctive among obese and overweight women with a wage penalty of 2.3–6.1%. [9] For the EU, it was found that women earned 3.3% and men 1.9% less hourly wages for every 10% increase in BMI above normal BMI [10]. Especially obese and overweight women in white-collar occupations are assumed to have lower resilience and poorer qualifications compared to women of normal weight [11]. Their income is lower, as wages peak at a BMI of 22 and decline with increasing bodyweight, which may also be an interpreted link of attractiveness and success. In contrast to this, it was found that men in blue-collar jobs even have higher wages when being overweight, interpreting a voluminous body as strength. [12]

**Fig. 1:**
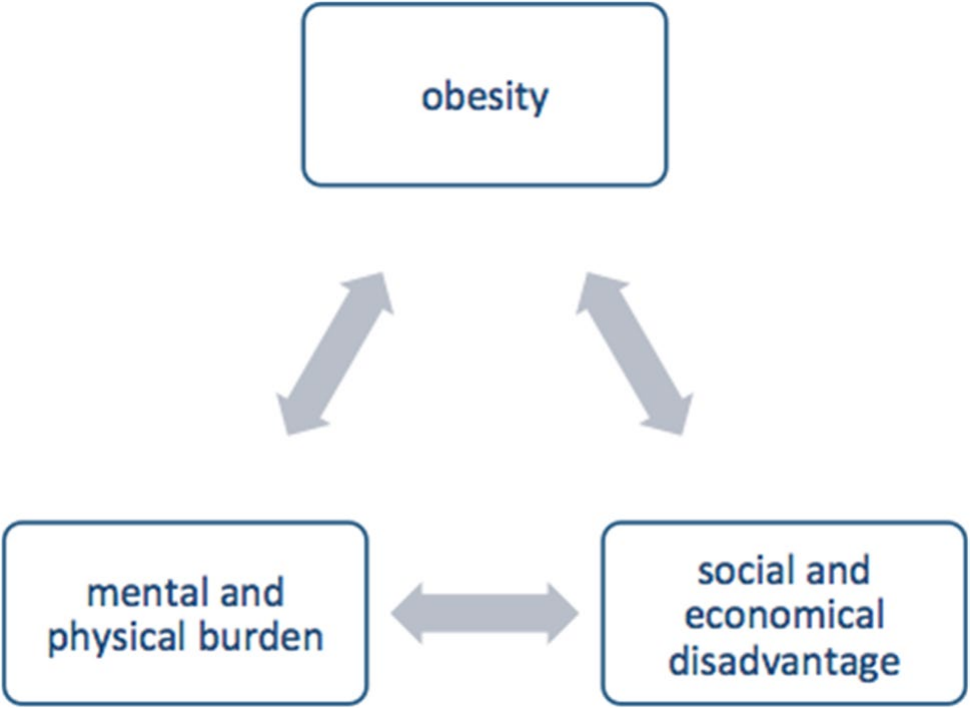
An overview about the resulting vicious circle of social decline, physical and mental health problems and unemployment in people affected from obesity and unemployment

In Germany, 26% of long-term unemployed women and 23% of long-term unemployed men are obese. In contrast, 16% of employed men and 13% of women are obese. [13] Obesity is most commonly established among women in the “lower educational group”, while this is only true for men older than 45 years [3] (Fig. 1).

Obesity is highly relevant from a health economics point of view since the direct and indirect costs of obesity to the German health care system are extrapolated to approximately 13 billion Euros per year. Alone in 2003, almost 50,000 cases of disability were caused by obesity, leading to approximately 500,000 lost working years. [14] It has been expected that the total costs increase to more than 5 billion Euros per year by 2020 [15].

According to the German Bariatric Surgery Registry, the most commonly performed bariatric interventions in Germany are the RYGB (Roux-en-Y gastric bypass) (38.15%; 17,215 of 45,121) and the SG (sleeve gastrectomy) (35.00%; 15,795 of 45,121) during 2010–2015, in favor to the SG during the last years. Most of these operations were performed laparoscopic. Further surgery methods were gastric banding, duodenal switch with or without bileopancreatic diversion and other complex (revision) surgery methods. [16] A SG can be performed both as an isolated procedure or as the initial step of a multi-stage therapy. Especially in European countries, recently the SG is performed more frequently because of its low complication rate. The RYGB achieves greater excess weight loss with a slightly higher postoperative morbidity rate and approximately the same safety compared to SG (Table 1). [17]

**Table 1:** Overview of a comparison between SG and RYGB, based on S3-Leitlinie “Chirurgie der Adipositas und metabolischer Erkrankungen” (S3 guideline “Surgery of obesity and metabolic diseases”) [17] [18] [19] [20]

	Weight loss within 2 years % EWL	Diabetic remission within 2 years %	Mortality %	Postoperative morbidity %	Complication rate %	Other
SG	49–81	53.3	0	7–8	12.1	Not recommended for refractory GERD
	46.7	60	0.29–0.34			
RYGB	62.1–94.4	83	0	21	20.9	More dumping, internal hernias, nutrient deficiencies
	80.1	77	0.38–0.72			

This article focuses on the highly relevant topic of bariatric treatment in the context of the labor market reintegration and integration of unemployed people suffering from obesity. We aim to generate knowledge regarding the best appropriate procedure in terms of return to work after bariatric surgery.

## Methods

This survey aims to identify if there is a difference in return to work in people affected by unemployment and obesity depending on the selection of the bariatric procedure (SG vs. RYGB).

Primary outcome parameter is the binary employment state 1 year after surgery. The secondary outcome parameter is percentage of total weight loss (%TWL). The bariatric procedure is defined as an independent binary variable. As relevant third variables sex, age and vocational training were included.

The retrospective cohort study is based on secondary pseudonymized data, i.e. the StuDoQ|MBE data set of the DGAV—Deutsche Gesellschaft für Allgemein- und Viszeralchirurgie e.V. (German Society for General Surgery). Since 2015, clinical data of patients with metabolic and bariatric diseases who gave informed consent is registered online by participating hospitals with obesity centers.

The inclusion criteria are defined as:

- being unemployed at the time of the surgery
- undergoing the surgery as first bariatric procedure
- having participated in the first follow-up (after 1 year)
- being between 18 and 65 years old in order to focus on the working age population

All patients who met these criteria were included being 782.

Patients who were incapacitated to work at the time of the surgery were excluded ex-ante because information on the cause were lacking. Otherwise, this would lead to an omitted variable bias, while a comparability to labor market studies could not be established. Nevertheless, a separate analysis is done descriptively. Patients who were completely lost to follow-up or retired regularly between the surgery and their first follow-up were excluded as well.

The data is described using a 2 × 2 table, while it is analyzed using the corresponding odds ratio (OR) and the adjusted OR within a logistic regression model in which age in years (at the time of the surgery), sex (binary), vocational training and %TWL act as third variables. While a weight gain is regarded as a weight loss of zero, the %TWL is calculated as the weight difference in percentage before and 1 year after surgery. The vocational training is split up into dummy variables with completed vocational training being the reference category. The levels “unknown”, “ongoing” and “other” were put together resulting in the categories university degree, complete vocational training and vocationally non-skilled. Significance is defined as *p* values < 0.05. For the logistic regression model’s coefficients, the Wald test is used. The model’s fit is assessed using McFadden’s pseudo *R*^2^. A potential moderation of the surgery technique’s influence on return to work is assessed by integrating an interaction term between sex and surgery technique. Additionally, the outcome employment after 2 years is described in relation to the bariatric procedure.

## Results

Of the surveyed patients, 25% were male, 75% female. The average age at the time of the surgery was 41.59 +  − 11.81 years; 41.51% received a RYGB, 58.49% a SG. While 3% graduated at the university and 35% completed vocational training, 35% are occupationally non-skilled and 26% reached other degrees (like in former East Germany).

The %TWL was 31.84 +  − 9.34%. Two patients actually gained weight; both of them received a SG. %TWL for SG is 31.16 +  − 9.97%, while it is 32.79 +  − 8.29% for RYGB.

39.39% of the patients with SG versus 33.85% with RYGB reached a return to work. Hence, the odds ratio for return to work is at 1.27 (*p* = 0.11) non-significantly in favor of SG versus RYGB. The total percentages of the sample can be found in Table 2.

**Table 2:** Return to work after SG and RYGB

	Return to work	No return to work	
Sleeve gastrectomy	180 (23.02%)	277 (35.42%)	457 (58.44%)
Roux-en-Y gastric bypass	110 (14.07%)	215 (27.49%)	325 (41.56%)
	290 (37.08%)	492 (62.92%)	*n* = 782 (100%)

**Fig. 2:**
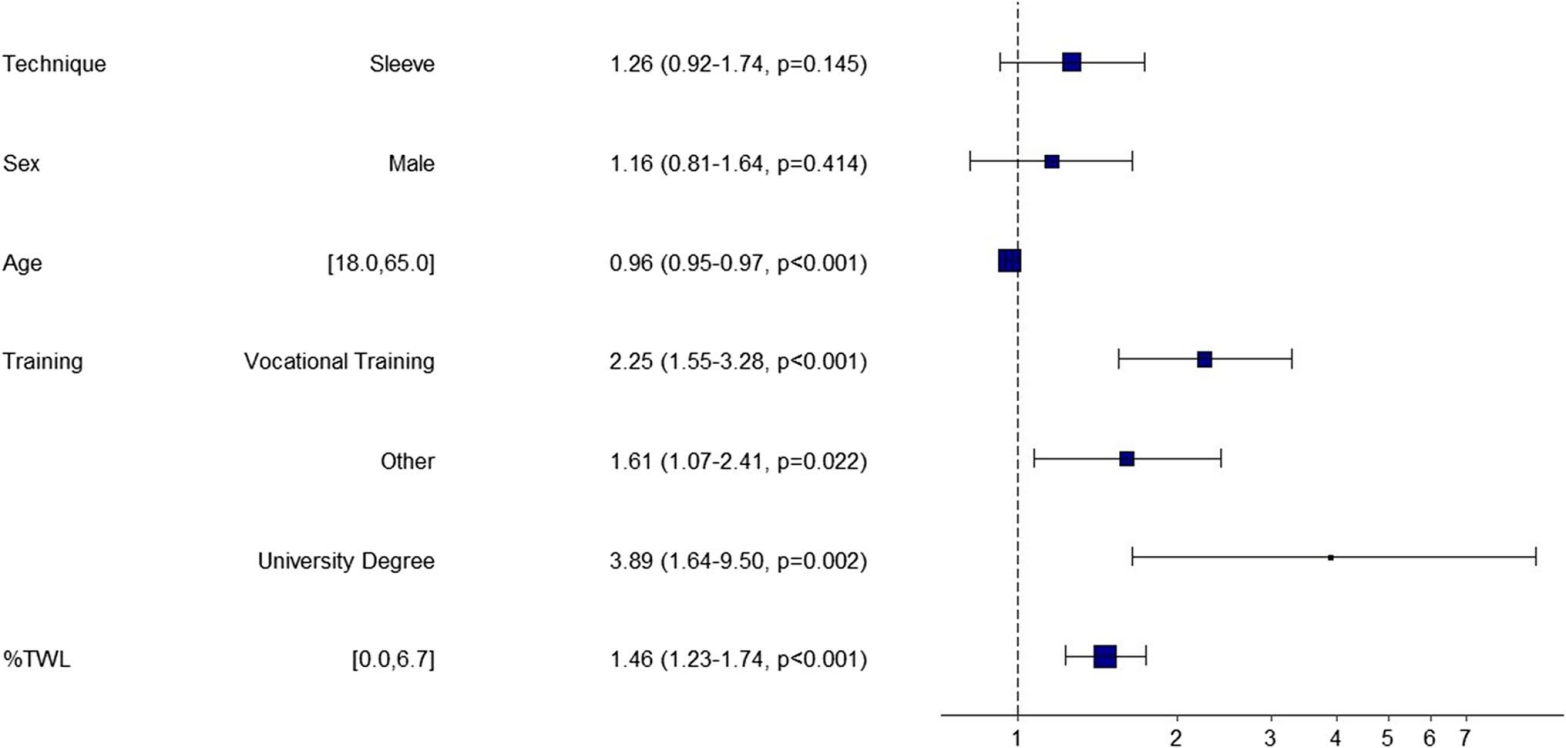
A graphical overview about the analyzed influences on return to work

The adjusted OR is 1.26 (*p* = 0.15), also indicating that there is no significant influence of the difference between these two surgery techniques on the outcome return to work after 1 year. Return to work after 1 year of surgery is affected significantly by age (negative relationship, *p* < 0.001), %TWL (positive relationship, *p* < 0.001) and a lack of vocational training (negative relationship, *p* < 0.001). For the logit coefficients of the logistic regression without interaction term, see Fig. 2. McFadden’s *R*^2^ is 0.09.

An interaction term between surgery technique and the patient’s sex is non-significantly positive (*β* = 0.04, *p* = 0.91) so that the influence of the surgery technique is not assumed to be moderated by the sex variable.

While the overall return to work rate is 37.08% after 1 year, eight participants were lost to follow-up. After 2 years, the overall return to work rate is 20.97%. Of those participants who reached a return to work after 1 year, 34.48% are still employed after another year. This is true for 33.33% of the SG and 36.36% of the RYGB patients.

Considering only patients who were incapacitated to work at the time of the surgery and excluded from the previous analysis (*n* = 195), their return to work rate after 1 year is 50.77%. For these patients undergoing SG, the chance of return to work is 50.48%. Here, the trend of an increased return to work rate after SGs compared to RYGBs cannot be observed (OR = 0.97, *p* = 0.93). However, there is a significant effect of sex on return to work among patients incapacitated to work in favor of men (OR = 0.382, *p* = 0.003).

## Discussion

Obesity is a main cause of social decline, physical and mental health problems as well as unemployment. This is accompanied by a major socio-economic impact considering direct and indirect costs. We aimed to evaluate the influence on bariatric procedures on unemployment and return to work. Therefore, we included data from patients who underwent SG or RYGB in 2015–2020 and were either unemployed (*n* = 782) or incapacitated to work (*n* = 195) at the time of surgery. There is no significant effect of the surgery technique on return to work, both in the bi- as well as in the multivariate analysis. In unemployed patients a slight trend of a higher return to work rate in patients who received SG compared to RYGB can be observed, which is not the case regarding patients incapacitated to work. While the overall sustainability of the success of the bariatric surgery is limited, there are only marginal differences regarding the sustainability of the chosen procedure in favor of the RYGB.

It can be seen that there is a correlation between age, vocational training and weight loss that influences return to work. In line with the information already elaborated, the data examined indicate that a high amount of weight loss and a high level of education seem to have a favorable influence on (re)employment. In our analysis, any vocational and educational degree has more influence on (re)employment than the selection of the bariatric procedure. Similarly, a correlation was found between younger age and a higher probability of return to work.

Sex has no influence on the relationship between the surgical procedure and return to work. However, among the patients who were incapacitated to work, men are more likely to return to work 1 year after surgery. This might be due to the fact that men in blue-collar jobs can potentially benefit from a BMI above normal regarding employment status and income, whereas women are more likely to face weight discrimination overall. [12]

In our study the average rate for return to work is more than one-third 1 year after obesity surgery. Comparing these findings to the overall “outflow rate” of the labor market (meaning the people who go from unemployment to employment) of 7.72% (15.2% of short-term and 3.3% of long-term unemployed) in Germany during 2018, [21] there seems to be a significant advantage of bariatric surgery in terms of return to work. However, a Swedish study showed that the initial advantage of bariatric surgery regarding employment status and wage disappeared on the long run [22].

Moreover, in Germany, there is a difference between “Erwerbslosigkeit” and “Arbeitslosigkeit”, both meaning unemployment and making a difference about marginal employment, whereas the latter does not consider a person with a marginal employment as being employed. The given outflow rate of the labor market of approximately 7.72% though does not take marginal employment into account which leads to a limitation regarding comparability with our findings. A further limitation is that the presence of relevant comorbidities is neither known nor included in this survey.

In the current status of research, there is a lack of evidence in terms of comparing the influence of surgical to non-surgical therapies of obesity on return to work in unemployed patients suffering from obesity. To address this issue, a prospective study in France and Great Britain is planned [23]. Further prospective data including patient-specific characteristics such as comorbidities, sex as well as long-term effects is desirable.

## Conclusion

People with obesity show a high rate of return to work after undergoing bariatric surgery. There is no significant difference between SG and RYGB although a tendency in favor of SG could be observed. Return to work, which is highly affected by age and training, is a multifactorial process, especially for people with obesity. Bariatric surgery may act as a key intervention for breaking the vicious circle of obesity and unemployment.

Facing labor market (re)integration, bariatric treatment should be made more accessible to persons suffering from chronic unemployment due to severe obesity.

## Abbreviations

BMI: Body mass index
DGAV: Deutsche Gesellschaft für Allgemein- und Viszeralchirurgie e.V. (German Society for General Surgery)
EU: European Union
GERD: Gastroesophageal reflux disease
i.e.: That is, id est
kg: Kilogram
OECD: The Organisation for Economic Co-operation and Development
OR: Odds ratio
RYGB: Roux-en-Y gastric bypass
SG: Sleeve gastrectomy
%TWL: Percentage of total weight loss
USA: United States of America
WHO: World Health Organization

## References

[1] WHO. Obesity and overweight [Internet]. [cited 2021 Feb 13]. Available from: https://www.who.int/news-room/fact-sheets/detail/obesity-and-overweight

[2] Mensink GBM, Schienkiewitz A, Haftenberger M, Lampert T, Ziese T, Scheidt-Nave C (2013). Übergewicht und Adipositas in Deutschland: Ergebnisse der Studie zur Gesundheit Erwachsener in Deutschland (DEGS1). Bundesgesundheitsbl.

[3] RKI. Übergewicht und Adipositas bei Erwachsenen in Deutschland. RKI-Bib1 (Robert Koch-Institut); 2017 [cited 2021 Apr 4]; Available from: http://edoc.rki.de/docviews/abstract.php?lang=ger&id=5128

[4] Baumeister H, Härter M (2007). Mental disorders in patients with obesity in comparison with healthy probands. Int J Obes.

[5] Hauner H, Moss A, Berg A, Bischoff SC, Colombo-Benkmann M, Ellrott T (2014). Interdisziplinäre Leitlinie der Qualität S3 zur „Prävention und Therapie der Adipositas”: der Deutschen Adipositas-Gesellschaft e.V.; der Deutschen Diabetes Gesellschaft; der Deutschen Gesellschaft für Ernährung e.V.; der Deutschen Gesellschaft für Ernährungsmedizin e.V. Version 2.0 (April 2014); AWMF-Register Nr. 050-001. Adipositas - Ursachen, Folgeerkrankungen, Therapie..

[6] Larsson U, Karlsson J, Sullivan M (2002). Impact of overweight and obesity on health-related quality of life—a Swedish population study. Int J Obes Relat Metab Disord. England.

[7] Arbeitsunfähigkeit - Anteil der wichtigsten Krankheitsarten an den AU-Tagen bis 2019 [Internet]. Statista. [cited 2021 Jun 19]. Available from: https://de.statista.com/statistik/daten/studie/77239/umfrage/krankheit---hauptursachen-fuer-arbeitsunfaehigkeit/

[8] Devaux M, Sassi F. The labour market impacts of obesity, smoking, alcohol use and related chronic diseases [Internet]. 2015 Nov. Report No.: 86. Available from: https://www.oecd-ilibrary.org/social-issues-migration-health/the-labour-market-impacts-of-obesity-smoking-alcohol-use-and-related-chronic-diseases_5jrqcn5fpv0v-en

[9] Baum CL, Ford WF (2004). The wage effects of obesity: a longitudinal study. Health Econ.

[10] Brunello G, D’Hombres B (2007). Does body weight affect wages? Evidence from Europe. Econ Hum Biol Netherlands.

[11] Giel KE, Thiel A, Teufel M, Mayer J, Zipfel S (2010). Weight bias in work settings—a qualitative review. Obes Facts.

[12] Caliendo M, Gehrsitz M (2016). Obesity and the labor market: a fresh look at the weight penalty. Econ Hum Biol.

[13] Gesundheitsverhalten und Gesundheitsressoucen von Arbeitslosen - LZG.NRW [Internet]. [cited 2021 Apr 4]. Available from: https://www.lzg.nrw.de/ges_foerd/ges_chanc_gl/arbeitslosigkeit_gesundheit/gesundheitsverhalten/index.html

[14] Knoll K-P, Hauner H. Kosten der Adipositas in der Bundesrepublik Deutschland. Adipositas - Ursachen, Folgeerkrankungen, Therapie. Schattauer GmbH; 2008;02:204–10.

[15] Kosten der Adipositas in Deutschland – Adipositas Gesellschaft [Internet]. [cited 2021 Jun 19]. Available from: https://adipositas-gesellschaft.de/ueber-adipositas/kosten-der-adipositas-in-deutschland/

[16] Ärzteblatt DÄG Redaktion Deutsches. Bariatrische Chirurgie: Magenbypass bevorzugte Operation [Internet]. Deutsches Ärzteblatt. 2016 [cited 2021 Feb 13]. Available from: https://www.aerzteblatt.de/archiv/179375/Bariatrische-Chirurgie-Magenbypass-bevorzugte-Operation

[17] Deutsche Adipositas-Gesellschaft e.V. (DAG) Deutsche Diabetes Gesellschaft e.V. (DDG) Deutsche Gesellschaft für Ernährungsmedizin e.V. (DGEM) Deutsche Gesellschaft für Endoskopie und bildgebende Verfahren e.V. (DGE-BV) Deutsche Gesellschaft für Psychosomatische Medizin und und Ärztliche Psychotherapie e.V. (DGPM) Deutsche Gesellschaft der Plastischen, Rekonstruktiven und Ästhetischen Chirurgen e.V, (DGPRÄC) Deutsches Kollegium für Psychosomatische Medizin (DKPM) Verband der Diabetesberatungs-und Schulungsberufe In Deutschland e.V. (VDBD) BerufsVerband Oecotrophologie e.V. (VDOE) Adipositaschirurgie-Selbsthilfe-Deutschland e.V. S3-Leitlinie: Chirurgie der Adipositas und metabolischer Erkrankungen. Version 2.3 (Februar 2018) AWMF-Register Nr. 088-001 [Internet]. [cited 2021 Apr 7]. Available from: https://www.awmf.org/leitlinien/detail/ll/088-001.html

[18] Colquitt JL, Pickett K, Loveman E, Frampton GK. Surgery for weight loss in adults. Cochrane Database of Systematic Reviews [Internet]. John Wiley & Sons, Ltd; 2014 [cited 2021 Feb 28]; Available from: https://www.cochranelibrary.com/cdsr/doi/10.1002/14651858.CD003641.pub4/full 10.1002/14651858.CD003641.pub4 PMC9028049 25105982

[19] Trastulli S, Desiderio J, Guarino S, Cirocchi R, Scalercio V, Noya G (2013). Laparoscopic sleeve gastrectomy compared with other bariatric surgical procedures: a systematic review of randomized trials. Surg Obes Relat Dis United States.

[20] Chang S-H, Stoll CRT, Song J, Varela JE, Eagon CJ, Colditz GA (2014). The effectiveness and risks of bariatric surgery: an updated systematic review and meta-analysis, 2003–2012. JAMA Surg.

[21] Bersheim SS Lena Becher, Frank Oschmiansky, Sabrina. Daten und Fakten: Arbeitslosigkeit | bpb [Internet]. bpb.de. [cited 2021 Apr 16]. Available from: https://www.bpb.de/politik/innenpolitik/arbeitsmarktpolitik/305833/daten-und-fakten-arbeitslosigkeit

[22] Norrbäck M, Neovius M, Ottosson J, Näslund I, Bruze G (2021). Earnings and employment for women after bariatric surgery: a matched cohort study. Int J Obesity Nature Publishing Group.

[23] Amenyah SD, Murphy J, Fenge L-A (2021). Evaluation of a health-related intervention to reduce overweight, obesity and increase employment in France and the United Kingdom: a mixed-methods realist evaluation protocol. BMC Public Health.

